# Consequences of the reduction of the Photosystem II antenna size on the light acclimation capacity of *Arabidopsis thaliana*

**DOI:** 10.1111/pce.13701

**Published:** 2020-02-05

**Authors:** Ludwik W. Bielczynski, Gert Schansker, Roberta Croce

**Affiliations:** ^1^ Biophysics of Photosynthesis/Energy, Faculty of Sciences, Department of Physics and Astronomy VU University Amsterdam Amsterdam The Netherlands

**Keywords:** antenna size reduction, dLhcb2, light acclimation, long‐term acclimation, photoprotection, photosynthesis, photosystem II antenna size, short‐term acclimation

## Abstract

In several systems, from plant's canopy to algal bioreactors, the decrease of the antenna size has been proposed as a strategy to increase the photosynthetic efficiency. However, still little is known about possible secondary effects of such modifications. This is particularly relevant because the modulation of the antenna size is one of the most important light acclimation responses in photosynthetic organisms. In our study, we used an *Arabidopsis thaliana* mutant (*dLhcb2*), which has a 60% decrease of Lhcb1 and Lhcb2, the two main components of the major Photosystem II antenna complex. We show that the mutant maintains the photosynthetic and photoprotective capacity of the Wild Type (WT) and adapts to different light conditions by remodelling its photosynthetic apparatus, but the regulatory mechanism differs from that of the WT. Surprisingly, it does not compensate for the decreased light‐harvesting capacity by increasing other pigment‐protein complexes. Instead, it lowers the ratio of the cytochrome b_6_f and ATP synthase to the photosystems, regulating linear electron flow and maintaining the photosynthetic control at the level of these complexes as in the WT. We show that targeting the reduction of two specific antenna proteins, Lhcb1 and Lhcb2, represents a viable solution to obtain plants with a truncated antenna size, which still maintain the capacity to acclimate to different light conditions.

## Abstract 2

Plants with reduced light‐harvesting complexes II (LHCII) content adapt to different light conditions using strategies different from WT plants, without modulating the pigment content. The reduction of LHCII can thus be a viable strategy to increase light‐use efficiency in the canopy.

## 1 INTRODUCTION

To meet the demand of the growing world population, the food production needs to be doubled until the end of this century, knowing that there are scarce prospects for the expansion of the cultivatable area (UN, 2015; Kline et al., 2017). To reach this goal, an increase in photosynthetic capacity of crops is needed (reviewed in Long, Marshall‐Colon, & Zhu, 2015).

A theoretical upper limit for the operational efficiency of plant photosynthesis from light capture to carbohydrate synthesis has been calculated to be about 4.6% for C_3_ plants (Zhu, Long, & Ort, 2008; Zhu, Long, & Ort, 2010). A decrease of the light‐harvesting capacity of the plants has been proposed as one of the possible strategies to maximize photosynthetic efficiency. It enables a more even distribution of light within the canopy, decreasing energy dissipation from the over‐illuminated top leaf layers and increasing the fraction of light energy reaching the under‐illuminated leaf layers deeper in the canopy (Melis, 2009; Ort et al., 2015; Ort, Zhu, & Melis, 2011; Song, Wang, Qu, Ort, & Zhu, 2017). The same principle can be applied to bioreactors, where a smaller antenna size of the photosystems decreases the over‐absorption of light by the cells closest to the light source, enabling deeper sunlight penetration into the culture. An increase of biomass production in mutants with truncated antenna size has been already validated for cyanobacteria (Kirst, Formighieri, & Melis, 2014 ; Nakajima & Ueda, 1997 ; Nakajima & Ueda, 1999), *Chlamydomonas reinhardtii* (Kirst et al., 2012, b ; Mussgnug et al., 2007 ; Polle, Kanakagiri, & Melis, 2003), species of the genus *Botryococcus* (reviewed in Melis, 2012) and tobacco plants (Kirst et al., 2018 ; Kirst, Gabilly, Niyogi, Lemaux, & Melis, 2017).

In plants, the transformation of light energy into chemical energy is performed by four major transmembrane protein complexes embedded in the thylakoid membrane: Photosystem II (PSII), cytochrome b_6_f, Photosystem I (PSI), and ATP synthase. PSII and PSI are multiprotein complexes composed of pigment‐binding proteins involved in light harvesting (antennae) and charge separation (reaction centre [RC]).

The PSII RC complex is a heterodimer of the D1 and D2 proteins, binding six molecules of chlorophyll (Chl) *a* and two pheophytins (Umena, Kawakami, Shen, & Kamiya, 2011; Wei et al., 2016). In the core, the RC is surrounded by two inner antennae (CP43 and CP47), binding 16 and 13 Chls *a*, respectively (Umena et al., 2011). These pigments transfer the absorbed energy to the RC expanding its absorption cross section. Further from the core are located the minor antennae (CP24, CP26, and CP29, with 14 Chls *a + b* each), which, similarly to the inner antennae, absorb light and transfer it to the RC. In addition, they are the docking sites for the light‐harvesting complexes II (LHCII) trimers, which are composed of the Lhcb1–3 proteins, and bind 42 Chls *a + b* (Ballottari, Girardon, Dall'Osto, & Bassi, 2012). All these complexes together form the PSII supercomplex (Caffarri, Kouřil, Kereïche, Boekema, & Croce, 2009; Dekker & Boekema, 2005; Su et al., 2017). The number of LHCIIs bound per supercomplex depends on the growth light (GL) conditions (Anderson, Chow, & Park, 1995; Ballottari, Dall'Osto, Morosinotto, & Bassi, 2007; Bielczynski, Schansker, & Croce, 2016 ; Wientjes, Van Amerongen, & Croce, 2013a) permitting the adaptation of the absorption cross‐section of the photosystems to the availability of light.

Similar to PSII, PSI is a large pigment‐binding supercomplex composed of a core containing the RC and around 100 Chls *a* and a light‐harvesting complex I (LHCI) system composed of four Lhca proteins (Lhca1–4) binding in total another 56 Chls (Qin et al., 2015). In the light, a pool of LHCII is also associated with PSI (Wientjes, Drop, Kouřil, Boekema, & Croce, 2013; Wientjes, van Amerongen, & Croce, 2013b).

Cytochrome b_6_f mediates and regulates the electron transfer between PSII and PSI. It oxidizes plastoquinol (PQH_2_) to plastoquinone (PQ) releasing the protons in the lumen and reduces the electron carrier that links cytochrome b_6_f with PSI: Plastocyanin (PC). It regulates the redox state of PQ and PC and contributes to the creation of the Δ*pH* across the thylakoid membrane (reviewed in Schöttler, Tóth, Boulouis, & Kahlau, 2015). Due to the lumen‐pH‐dependent rate of reoxidation of PQH_2_ (the so‐called “photosynthetic control”), the electron flow (EF) through the complex changes substantially under different conditions (Joliot & Johnson, 2011; Stiehl & Witt, 1969).

Finally, the fourth complex, the ATP synthase, utilizes the proton‐motive force (pmf) to synthesize ATP. The process is highly regulated (Kanazawa & Kramer, 2002), and the dissipation rate of the pH gradient can influence the EF through photosynthetic control.

Truncated antenna mutants are available since a long time in several species (see Melis, 1991). The reduction of the antenna size can be obtained in several ways: (a) by targeting proteins involved in the import in the chloroplast (e.g., Kirst et al., 2018) or (b) in the assembly of the antennae proteins with pigments (e.g., Ghirardi, McCauley, & Melis, 1986; Hansson, Kannangara, Wettstein, & Hansson, 1999; Sakowska et al., 2018) or (c) by targeting individual LHCs (e.g., Andersson et al., 2003; Dall'Osto et al., 2017; Dall'Osto, Ünlü, Cazzaniga, & Van Amerongen, 2014; de Bianchi et al., 2011; de Bianchi, Dall'Osto, Tognon, Morosinotto, & Bassi, 2008; Pietrzykowska et al., 2014). Although all those methods produced plants with a truncated antenna, the effect on the biomass production varied (e.g., Kirst et al., 2018; Sakowska et al., 2018). A possible reason for these apparently contradictory results is that those mutations induce pleiotropic effects hindering the capacity of the plants to perform and/or regulate photosynthesis. For example, mutants lacking the antennae completely were shown to become damaged in high light (Ramel et al., 2013). Mutants lacking the minor antennae have a bad connection between PSII and the core, which decreases the PSII trapping efficiency (Dall'Osto et al., 2014; Van Oort et al., 2010). The partial reduction of the antenna achieved in an untargeted way also produced contrasting results (Kirst et al., 2017; Slattery, Vanloocke, Bernacchi, Zhu, & Ort, 2017), suggesting that the difference can be due to secondary effects induced by the mutation.

A way to get around these issues is to specifically target a protein directly composing the LHCIIs, which are located at the periphery of the supercomplex, and the absence of which should thus not influence the efficiency of energy delivery to the RC. However, because LHCII modulation is a major acclimation strategy of plants (Anderson et al., 1995; Ballottari et al., 2007; Bielczynski et al., 2016; Wientjes, Van Amerongen, & Croce, 2013a), it was unclear if the absence of a part of the LHCIIs would influence the acclimation capacity of the plant. To answer this question, we challenged a mutant having a smaller pool of LHCII (Andersson et al., 2003) to grow under different light conditions. Combining biochemical and functional measurements, we show that a targeted reduction of the LHCII pool is a viable strategy to decrease the antenna size without introducing secondary effects that negatively impact its performance.

## 2 MATERIALS AND METHODS

### 2.1 Plant material

*A. thaliana* (ecotype Col‐0) WT and *dLhcb2* seeds (transgenic line Lhcb2–12 from Arabidopsis Biological Resource Center described in Andersson et al., 2003) were sown on Murashige and Skoog medium agar plates. After 5–7 days, the seedlings were transplanted to final pots. Plants were grown for 6–7 weeks in growth chambers (AR‐36 L, Plant Climatics Percival) at 70% RH, 21°C, with a photoperiod of 8/16 hr (day/night) and under 200 and 600 μmol photons m^−2^ s^−1^ (GL200 and GL600, respectively). After 4 weeks, a set of plants from GL200 was transferred to 1800 μmol photons m^−2^ s^−1^ (FytoScope FS 3400, Photon Systems Instruments) for another 2 weeks (GL1800).

### 2.2 Leaf absorption measurements

Leaf absorption was measured using a Cary 4000 UV‐Vis spectrophotometer with a mounted integrating sphere. Each leaf was carefully placed to ensure the same light cross‐section was obtained and that the whole measuring beam was passing through the leaf.

### 2.3 Thylakoid isolation

The isolation procedure was described in Robinson, Sharp, and Yocum (1980) with modifications from Caffarri et al. (2009). The isolation was performed on over‐night dark‐adapted plants. Isolated thylakoid membranes were resuspended in 20‐mM HEPES, pH 7.5, 0.4‐M sorbitol 15‐mM NaCl, 5‐mM MgCl_2_ and stored until further use at −80°C, after rapid freezing in liquid nitrogen.

### 2.4 Pigment isolation

The Chl *a*/*b* ratio and Chl/carotenoid ratio were determined from the absorption spectra of 80% acetone extracts. The absorption spectra were fitted with the spectra of individual pigments in the same solvent, as described in Croce, Canino, Ros, and Bassi (2002). The quantification of different carotenoids was performed by HPLC using a System Gold 126 Solvent module and 168 Detector (Beckman Coulter) as described by Gilmore and Yamamoto (1991) with the modification reported in Xu, Tian, Kloz, and Croce (2015).

### 2.5 2D‐polyacrylamide gel electrophoresis

Blue native (BN)‐polyacrylamide gel electrophoresis (PAGE) was performed as described in Järvi, Suorsa, Paakkarinen, and Aro (2011) with modifications described in Bielczynski et al. (2016). Two types of resolving gels were used depending on the scope of the analysis, 4–12.5% or 5–12.5%. For the 2D‐PAGE, we used the Tris‐tricine‐sodium dodecyl sulphate (SDS) PAGE system (Schägger, 2006). We have estimated the amount photosynthetic complexes by summing the corresponding Integrated Optical Density (IODs) of reference proteins PSII: CP43 and CP47; PSI: PsaA and PsaB; cytochrome b_6_f: PetB, PetC, and PetD; ATP synthase: AtpA and AtpB.

### 2.6 Functional antenna size of PSII and PSI

PSII functional antenna size measurements were performed as in Dinç, Ceppi, Tóth, Bottka, and Schansker (2012) with the modifications described in Bielczynski et al. (2016). PSI functional antenna size was measured as described by Takahashi, Clowez, Wollman, Vallon, and Rappaport (2013) with some modifications. After infiltrating the leaves with 200‐*μ*M DCMU (3‐(3,4‐dichlorophenyl)‐1,1‐dimethylurea) and 1‐mM HA and effectively inhibiting the PSII (F_V_/F_M_ < 0.1), the changes in the absorption at 520–546 nm, from electrochromic shift (ECS) generated by only PSI RCs, were measured in continuous red light (emission peak at 630 nm) of 300 μmol photons m^−2^ s^−1^ with a JTS‐10 (BioLogic). The slope of the first 10 ms of illumination was used to estimate the functional antenna size of PSI.

### 2.7 ATP synthase activity

The ATP synthase activity was monitored by measuring the proton conductivity as in Cruz, Sacksteder, Kanazawa, and Kramer (2001). We used a JTS‐10 (BioLogic) and measured the ECS signal decay (520–546 nm) following a 50 s red light (630 nm) illumination (300 μmol photons m^−2^ s^−1^). Subsequently, the signal was double normalized. The quasi‐steady‐state level at the end of the illumination period was defined as zero and the signal level 400 ms after the light‐to‐dark transition was defined as one.

### 2.8 Cytochrome b_6_f resistance measurements

Cytochrome b_6_f resistance (Harbinson & Hedley, 1989; Harbinson & Hedley, 1993; Ott, Clarke, Birks, & Johnson, 1999) was measured with a Dual‐PAM‐100 fluorometer (Walz). The redox changes of P700 were followed by recording the difference between the absorption changes at 830 and 875 nm. At the beginning of the measurement, *ΔA*_*max*_ was measured from the absorption changes before and during the SP according to Klughammer and Schreiber (1994). After 8‐min illumination with red light (630 nm) of 1,000 μmol photons m^−2^ s^−1^, the PQ pool was mostly reduced, and the PSI RCs were mostly oxidized. After switching off the light the electrons were transferred from the PQ pool via cytochrome b_6_f to oxidized PC and P700, the RC of PSI. Because re‐oxidation of PQH_2_ by cytochrome b_6_f is the rate‐limiting step, the rate of re‐reduction of P700 becomes an estimate of the cytochrome b_6_f resistance. The P700^+^ re‐reduction kinetics was normalized to the absorption minimum at 50 ms after the AL was switched off, and rescaled to *ΔA*_*max*_, to allow a comparison of the kinetics of the different measurements.

### 2.9 Quenching analysis

Chl fluorescence and P700 absorption (830–875 nm) changes were measured simultaneously on intact leaves, at room temperature, with a DUAL‐PAM‐100 fluorometer (Walz). The saturating pulse (SP) intensity was 5,000 μmol photons m^−2^ s^−1^ and the measuring light (ML) intensity 3 μmol photons m^−2^ s^−1^. The *F*_*0*_ was assessed with a weak ML after dark acclimation and *F*_*M*_ as the peak‐value during a 500‐ms long SP. After a subsequent 15‐s far‐red (FR) illumination (89 μmol photons m^−2^ s^−1^), the *ΔA*_*max*_ was measured from the absorption changes before and during the SP according to Klughammer and Schreiber (1994). To characterize the dark‐to‐light transition, the plants were light acclimated for 8 min with a selected actinic light (AL) intensity (200, 600, or 1,000 μmol photons m^−2^ s^−1^), where every 20 s, an SP with a 5 s FR pre‐illumination was applied to the sample to determine *F*_*M*_ *′* and a 2‐s period of darkness to measure and calculate *ΔA*_*sat*_. Before each SP, *F*_*t*_ was measured (ML + AL). After switching off AL, the light‐to‐dark transition was recorded. During the recovery period, every 50 s, an SP was applied to the sample, and the same parameters were determined. From the fluorescence measurements, *F*_*V*_ */F*_*M*_, nonphotochemical quenching (*NPQ*), *Φ*_*PSII*_, *Φ*_*NPQ*_, and *Φ*_*NO*_ were calculated, based on Butler and Kitajima (1975), Genty, Briantais, and Baker (1989) and Klughammer and Schreiber (2008), as follows

(1) $$F_{V}/F_{M}=\frac{F_{M}-F_{0}}{F_{M}}.$$

(2) $$\mathrm{NPQ}=\frac{F_{M}-F_{M}^{\prime }}{F_{M}^{\prime }}.$$

(3) $$\Phi_{\text{PSII}}=\frac{F_{M}^{\prime }-F_{t}}{F_{M}^{\prime }}.$$

(4) $$\Phi_{\mathrm{NPQ}}=\frac{F_{t}}{F_{m}^{\prime }}-\frac{F_{t}}{F_{M}}.$$

(5) $$\Phi_{\mathrm{NO}}=\frac{F_{t}}{F_{m}^{\prime }}.$$

### 2.10 Irradiance curve

During irradiance curve measurements, as well as during quenching analysis measurements, we used two‐channel mode and the settings for ML and SP were kept the same. However, dark‐acclimated plants were first pre‐illuminated for 10 min with 53 μmol photons m^−2^ s^−1^ AL to activate Ferredoxin NADP+ oxidoreductase (FNR). Afterward, the plants were acclimated for 60 s to 10 different AL intensities, in the range from 0 to 1,288 μmol photons m^−2^ s^−1^. After each acclimation period, an SP was applied to the samples, and the same parameters as during the quenching analysis were determined. Additionally, *ETR*_*II*_, *qP*, and *P700* ^*red*^ were calculated. The *qP* parameter (Genty et al., 1989) is based on the *qQ* parameter from Schreiber, Schliwa, and Bilger (1986).

(6) $$\mathrm{qP}=\frac{F_{M}^{\prime }-F_{t}}{F_{M}^{\prime }-F_{0}^{\prime }},$$

where *F*_*0*_ *′* is calculated according to the Oxborough and Baker (1997) approximation.

(7) $$F_{0}^{\prime }=\frac{F_{0}}{F_{V}/F_{M}+F_{0}/F_{M}^{\prime }}.$$

As for *ETR*_*II*_, we calculated it according to Genty et al. (1989) with some modifications:

(8) $${\mathrm{ETR}}_{\mathrm{II}}={\text{IδαΦ}}_{\text{PSII}},$$

where I is the incident light intensity, δ is the correction factor for the fraction of light that did not reach PSII as it got absorbed by PSI and α is the correction for the antenna size of PSII.

The *P700* ^*red*^ is calculated in the following manner:

(9) $$P700^{\mathrm{red}}=\Phi_{\mathrm{PSI}}+\Phi_{\mathrm{NA}},$$

where *Φ*_*PSI*_ and *Φ*_*NA*_ are the quantum yields of PSI and acceptor side limitation of PSI, respectively.

## 3 RESULTS

### 3.1 WT and *dLhcb2* comparison

When grown under standard conditions (GL200), the *dLhcb2* plants showed a higher Chl *a*/*b* and a lower Chl/Car ratio than the WT plants. The mutant also contained around 20% less Chls per fresh weight than the WT; it had a slightly lower F_V_/F_M_ value and a lower NPQ level. These data are similar to those observed before for the same mutant (Andersson et al., 2003).

### 3.2 Thylakoid membrane composition

**Figure 1:**
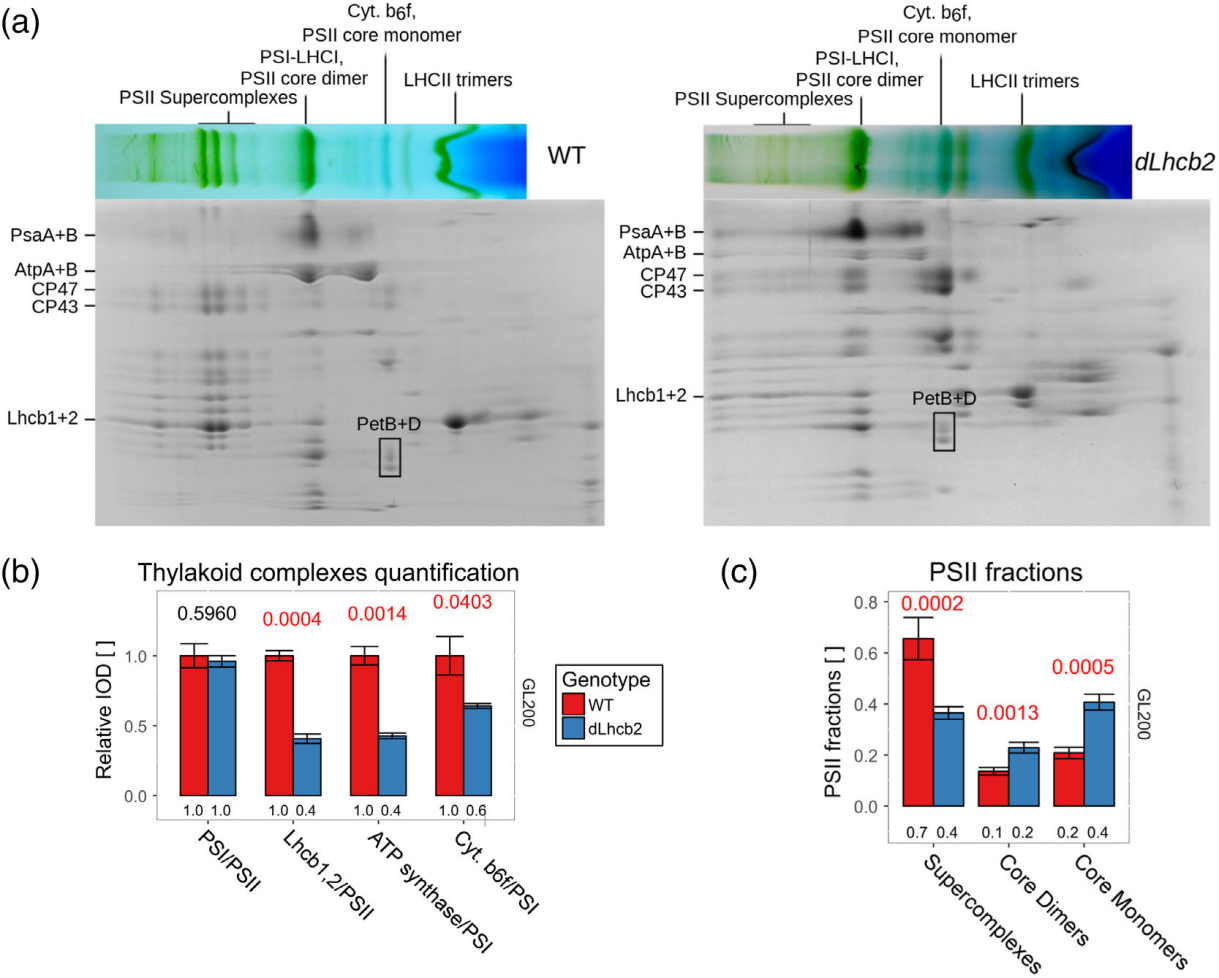
2D‐polyacrylamide gel electrophoresis (PAGE) analysis of *dLhcb2* and WT plants. (a) 2D‐PAGE on thylakoid membranes from *dLhcb2* and WT (right and left panel, respectively) grown under GL200. On top of each sodium dodecyl sulphate‐PAGE, the strip excised from a BN‐PAGE is shown. On the side, the labels corresponding to specific groups of protein dots are indicated. (b) Relative quantification of the photosynthetic complexes and the antenna size of PSII from a 2D‐PAGE based on three independent repetitions (*n* = 3) on WT and *dLhcb2* (red and blue fill, respectively) grown under GL200. The data were normalized to PSII or PSI and to WT. Numbers above the bars are the *p* ‐values of ANOVA's *F* test on a specific group. Red signifies the rejection of the null hypothesis that the response to all growth light intensities is the same (*α <* 0.05), black its acceptance. IOD, Integrated Optical Density; LHC, light‐harvesting complex; PSI, Photosystem I; PSII, Photosystem II; GL, growth light

In the mutant, the silencing of Lhcb2 should results in a decrease in the absorption cross‐section of PSII. However, it was shown by Andersson et al. (2003) that the CO_2_ assimilation rate is maintained at a similar level as in the WT. Such a result suggests that changes in the composition and/or performance of the complexes involved in photosynthesis compensate for the change in PSII antenna size. To validate this hypothesis, we quantified the major photosynthetic complexes responsible for the light phase of photosynthesis by performing a 2D‐PAGE analysis (Figure 1).

To keep the PSII supercomplexes intact, we solubilized the thylakoid membranes with a mild detergent (1% α‐DDM). The solubilized complexes were separated in their native state on a BN‐PAGE. An SDS–PAGE in the second dimension was used to separate the proteins of each complex (Figure 1a). The proteins were identified on the basis of previous work (Andaluz et al., 2006; Aro et al., 2005; Bielczynski et al., 2016; Caffarri et al., 2009; Takabayashi et al., 2013). To quantify PSII, we used the sum of the dots corresponding to CP43 and CP47. As a reference for PSI, we used PsaA and PsaB, for cytochrome b_6_f PetB and PetD, and for the ATP synthase AtpA and AtpB. To get a general estimate of the antenna size of PSII, we used the sum of the Lhcb1 and Lhcb2 dots. Due to a similar MW of both proteins, they overlap in the gel, and as a consequence, we cannot discriminate between them.

The PSI/PSII (core) ratio was the same in the WT and in the mutant (*p* = .6), whereas the Lhcb1 and Lhcb2 content dropped to 40% of the WT value in the mutant (*p* < .001; Figure 1b). Surprisingly, the amount of these two proteins in the mutant was far higher than reported previously by Andersson et al. (2003) and Ruban et al. (2003), probably due to the instability of the RNA silencing. Interestingly, we also observed a decrease of cytochrome b_6_f and ATP synthase as compared with the WT (to 60% and 80%, respectively, with *p* < .05; Figure 1b).

In agreement with the decrease in the content of Lhcb1 and Lhcb2, the native gel showed that the amount of PSII supercomplexes was lower in the mutant. Indeed, in the WT, most of the PSII was organized in supercomplexes (~70%) and the rest in the form of core monomers and dimers (Figure 1c), in *dLhcb2*, those proportions were inverted: <40% of PSII was in the form of supercomplexes, >40% in the form of core monomers, and around 20% in the form of core dimers. However, the protein composition of the supercomplexes was the same in WT and mutant and contained the antennae CP24, CP26, CP29, Lhcb3, and Lhcb1/Lhcb2.

### 3.3 PSII antenna composition

**Figure 2:**
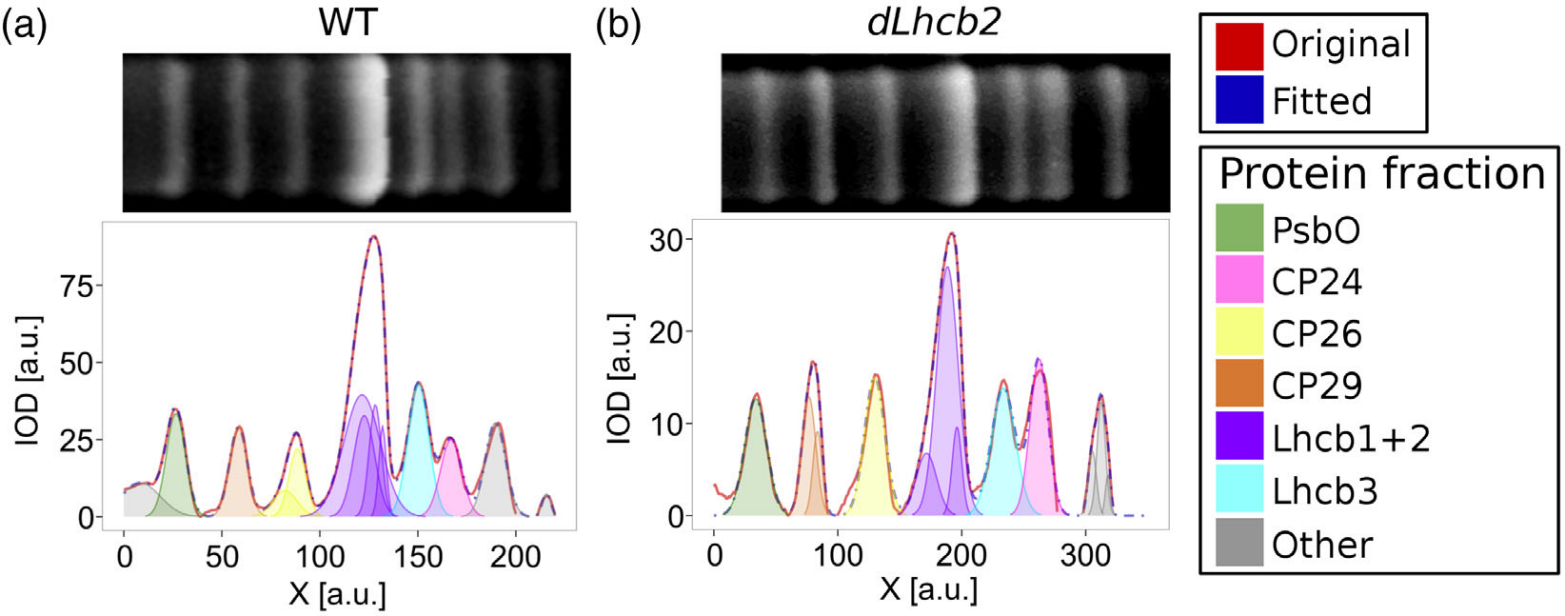
Thylakoid membrane proteins from WT and *dLhcb2* separated using TT sodium dodecyl sulphate–polyacrylamide gel electrophoresis (SDS–PAGE). Top, TT‐SDS PAGE lane fragments of WT and *dLhcb2* (Panels a and b, respectively) containing PSII antenna proteins. Bottom, IOD profiles of the lane fragment (solid red trace), with a fitted Gaussian sum (dash‐dotted blue trace). Specific Gaussians corresponding to the PsbO, CP24, CP26, CP29, Lhcb1 + 2, Lhcb3, and other proteins are shown (green, pink, yellow, orange, purple, cyan, and grey, respectively). LHC, light‐harvesting complex; WT, Wild Type [Color figure can be viewed at https://wileyonlinelibrary.com]

It was previously shown that the absence of Lhcb1 and Lhcb2 was partially compensated by an increase of CP26 (Ruban et al., 2003). To determine the PSII antenna composition, we separated the thylakoid membrane proteins in 1D‐PAGE (TT‐SDS PAGE; Figure 2). As some of the protein bands were slightly overlapping in the gel, we used nonlinear least squares to fit the integrated optical density of the lanes with a sum of Gaussians.

This analysis confirms the decrease of Lhcb1 and Lhcb2 in *dLhcb2* as compared with the WT (Table 2). We verified it while normalizing the number of antenna proteins to CP29: Lhcb3 also decreased, but all three major components of LHCII were still present in the plants. The decrease in Lhcb1 and Lhcb2 was not compensated by an increase of CP26 because this protein did not differ significantly in the WT and in the *dLhcb2* mutant.

### 3.4 Energy partitioning in PSII

**Figure 3:**
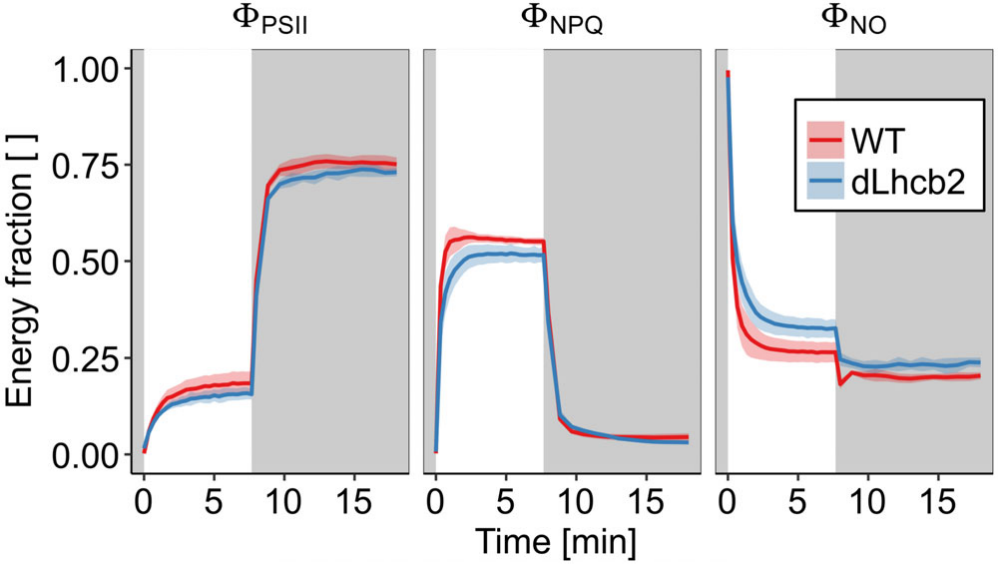
Energy partitioning during quenching analysis. During NPQ, induction fluorescence parameters were measured on intact leaves of Wild Type (WT) and *dLhcb2* (red and blue traces, respectively), grown under GL200, and Φ_PSII_, Φ_NPQ_, and Φ_NO_ were estimated (left, middle and right panel, respectively). The average of the measurements performed on five different plants (*n* = 5) is represented as a solid line and the shadow is the standard deviation. With the grey and white background, respectively, the periods of darkness and illumination with AL of 1,000 μmol of photons m^−2^ s^−1^ are shown. NPQ, nonphotochemical quenching; PSII, Photosystem II [Color figure can be viewed at https://wileyonlinelibrary.com]

To observe how the changes in the protein composition influenced the photosynthetic and the photoprotective capacity of the mutant, we performed a quenching analysis (Figure 3). The effective quantum yield of PSII (Φ_PSII_) was hardly affected in the mutant. The fast induction phase of NPQ was identical to that of the WT whereas the second phase was slightly slower in the mutant (Figure 3a). A small increase in the amount of energy directed to Φ_NO_ in comparison with the WT was also observed, suggesting that the mutant can be slightly less photoprotected.

### 3.5 Light acclimation

#### 3.5.1 General comparison

**Figure 4:**
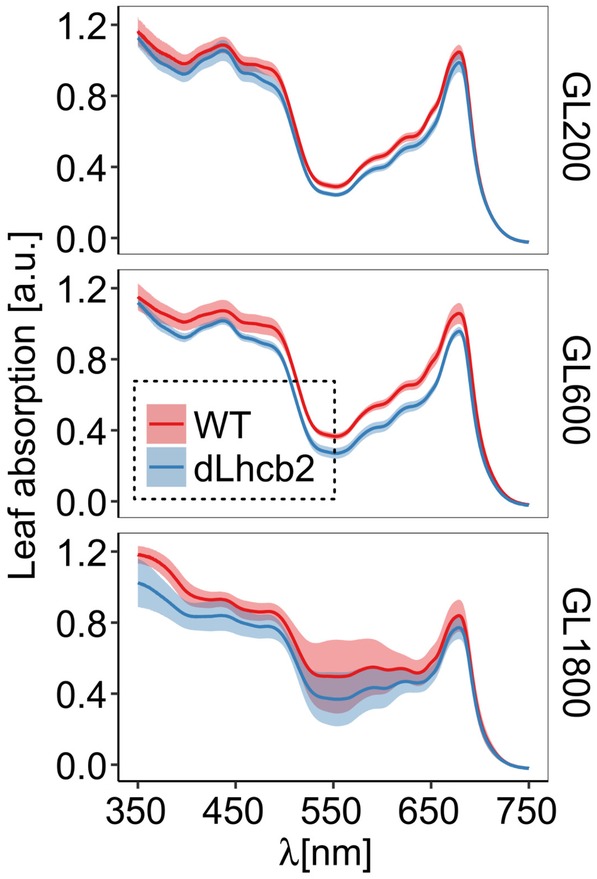
Leaf absorption of Wild Type (WT) and *dLhcb2* plants long‐term acclimated to different light intensities. Leaf absorption spectra in the visible range (350–750 nm) of WT and *dLhcb2* (red and blue traces, respectively) grown under GL200, GL600, and GL1800 (top, middle, and bottom panels, respectively), measured on different leaves coming from five different plants (*n* = 5) grown under previously mentioned conditions. The standard deviation is shown as shadows around the traces. GL, growth light; LHC, light‐harvesting complex [Color figure can be viewed at https://wileyonlinelibrary.com]

The data above suggest that the reduction of Lhcb1 and Lhcb2 may negatively influence the photoprotective capacity of the plant. However, a reduction of the antenna size and especially a decrease of Lhcb1 and Lhcb2 as it is the case in this mutant is a natural strategy used by plants for acclimation to high light (Ballottari et al., 2007; Kouřil, Wientjes, Bultema, Croce, & Boekema, 2013; Wientjes, Van Amerongen, & Croce, 2013a). We then tested the performances of the mutants in higher light conditions, comparing plants grown under 200, 600, and 1,800 μmol of photons m^−2^ s^−1^. The mutant was slightly paler compared with the WT, in agreement with a decreased Chl content (Figure 4 and Table 3).

### 3.6 Pigment composition

In the WT, the exposure to higher light intensities led to a decrease in Chl content. In *dLhcb2*, the concentration of Chls still decreased with increasing GL intensity (Table 3), although less than in the WT.

The Chl *a/b* ratio increased in the WT at higher light intensities as observed before (Bielczynski et al., 2016; Wientjes, Van Amerongen, & Croce, 2013a), but did not change in the mutant. This result suggests that the mutant cannot regulate the antenna size, but only the concentration of Chls. At GL1800, the Chls/fresh weight was the same in WT and mutant, but the Chl *a/b* ratio differed suggesting a different composition of the membranes.

The Chl/Car ratio decreased with increasing GL in both mutant and WT, mainly due to a decrease in the Chl content. The changes for most of the carotenoids were similar in WT and mutant, except for lutein, which increased substantially in the mutant in high light. This is surprising because lutein in plants is associated with the antennae and LHCII in particular (Ballottari et al., 2012; Wei et al., 2016) and thus its content in the mutant is expected to be lower. It is therefore likely that part of the lutein in the mutant is free in the membrane.

### 3.7 Thylakoid membrane composition

**Figure 5:**
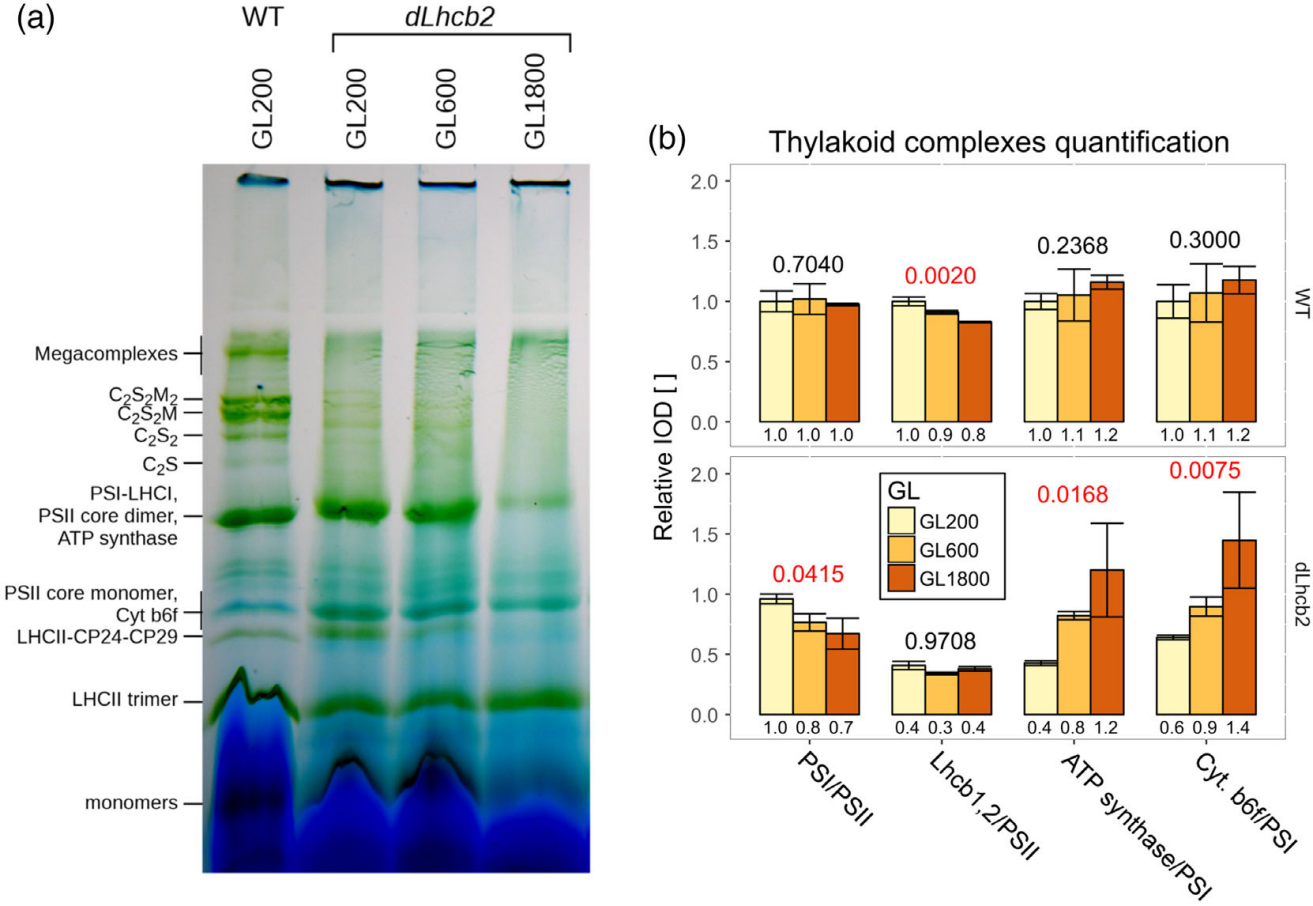
Quantification of thylakoid complexes of *dLhcb2* and Wild Type (WT) plants long‐term acclimated to different light intensities. (a) The BN‐polyacrylamide gel electrophoresis of thylakoid membranes isolated from *dLhcb2* plants grown under GL200, GL600, and GL1800, solubilized with 1% *n‐dodecyl‐α‐D‐maltoside (α*‐DDM). WT grown under GL200 was used as a control. (b) Relative quantification of the light phase photosynthetic complexes from a 2D‐polyacrylamide gel electrophoresis based on three independent repetitions (*n* = 3) on WT and *dLhcb2* (Panels a and b, respectively) grown under GL200, GL600, and GL1800 (yellow, orange, and brown fill, respectively). The data were normalized to PSII and to WT GL200. Numbers above the bars are the p‐values of ANOVA's *F* test on a specific group. Red signifies rejection of the null hypothesis that the response to all growth lights is the same (α < .05), black its acceptance. GL, growth light; IOD, integrated optical density; LHC, light‐harvesting complex; PSI, Photosystem I; PSII, Photosystem II [Color figure can be viewed at https://wileyonlinelibrary.com]

To check for changes in the composition of the thylakoid membrane of the mutant upon acclimation to a different light intensity, we performed BN‐PAGE (Figure 5a) followed by a 2D‐PAGE analysis (Figure S1, supporting information) as described above.

For the WT, it was shown before (Bielczynski et al., 2016) that the amount of Lhcb1 and Lhcb2 *per* PSII core decreased when the plants were grown under increasing light intensities (*p* < .05). At variance with this, in *dLhcb2*, under all light intensities, the amount of Lhcb1 and Lhcb2 *per* PSII core remained the same (*p* = .97), and it was always lower than in the WT, even at very high light intensities.

In the WT, the PSI/PSII ratio and the amount of ATP synthase and cytochrome b_6_f relative to PSI did not differ in plants grown under different light intensities. In the mutant instead, a decrease in the PSI/PSII ratio and a large increase in the ATP synthase and cytochrome b_6_f per PSI were visible in high light (GL1800). The differences observed for the ATP synthase and cytochrome b_6_f are significant, even when the relative amounts are renormalized to PSII.

### 3.8 Functional antenna size of the photosystems

**Figure 6:**
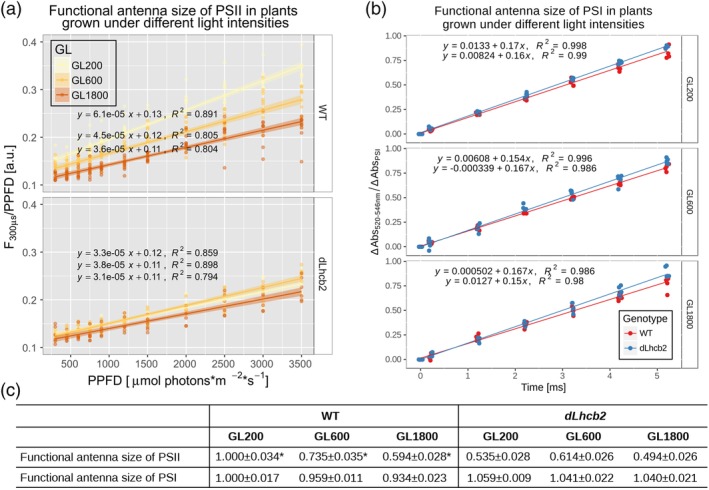
Functional antenna size of PSII and PSI. (a) Relationship between fluorescence intensity at 300 μs normalized to PPFD, and PPFD fitted with linear regression. The fitted lines with their standard error are shown as lines with shadows. Individual data points are from the WT (data from Bielczynski et al., 2016) and *dLhcb2* (top and bottom panels respectively) plants grown under GL200, GL600, and GL1800 (yellow, orange, and brown, respectively). The functional antenna size of PSII was measured on 10 different leaves (*n* = 10). The asterisk indicates data from an experiment carried out in parallel on WT described in Bielczynski et al. (2016). (b) ECS rise during the illumination coming from PSI, after normalization to the amount of PSI. The functional antenna size of PSI was measured on five different leaves (*n* = 5) from WT and *dLhcb2* plants (top and bottom panel, respectively) grown under GL200, GL600, and GL1800 (yellow, orange, and brown, respectively). (c) The functional antenna size of PSI corresponds to the slope and standard error of a fit of the ECS rise during the illumination period, due to charge separations in PSI. The functional antenna size of PSII corresponds to the slope and standard error of a fit of the normalized fluorescence intensity at 300 μs against PPFD relationship. ECS, electrochromic shift; GL, growth light; PSI, Photosystem I; PSII, Photosystem II; PPFD, Photosynthetic Photon Flux Density; WT, Wild Type [Color figure can be viewed at https://wileyonlinelibrary.com]

Because no changes in the LHCII/PSII ratio at the protein level in the mutant grown in different light intensities were observed, we then looked for possible changes in the functional antenna size (Figure 6b). The PSII functional antenna size of the mutant was around 40–50% smaller than that of the WT at GL200 (Figure 6c). Moreover, although in the WT, the functional antenna size decreased with light intensity, in the mutant, the differences were small (~10%), in line with the protein composition.

We also tested the PSI functional antenna size in WT and mutant after acclimation to the three different light intensities (Figure 6a). The measurements were performed on dark‐acclimated samples when the plants were in State I (Pesaresi et al., 2009; Tikkanen et al., 2008; Wientjes, Drop, et al., 2013; Wientjes, van Amerongen, & Croce, 2013b). In the WT, the functional antenna size of PSI remained very similar, changing only by about 7% (decreasing slightly, when grown under increasing light intensities; Figure 6c). In the mutant, no changes were detected.

### 3.9 Cytochrome b_6_f resistance

**Figure 7:**
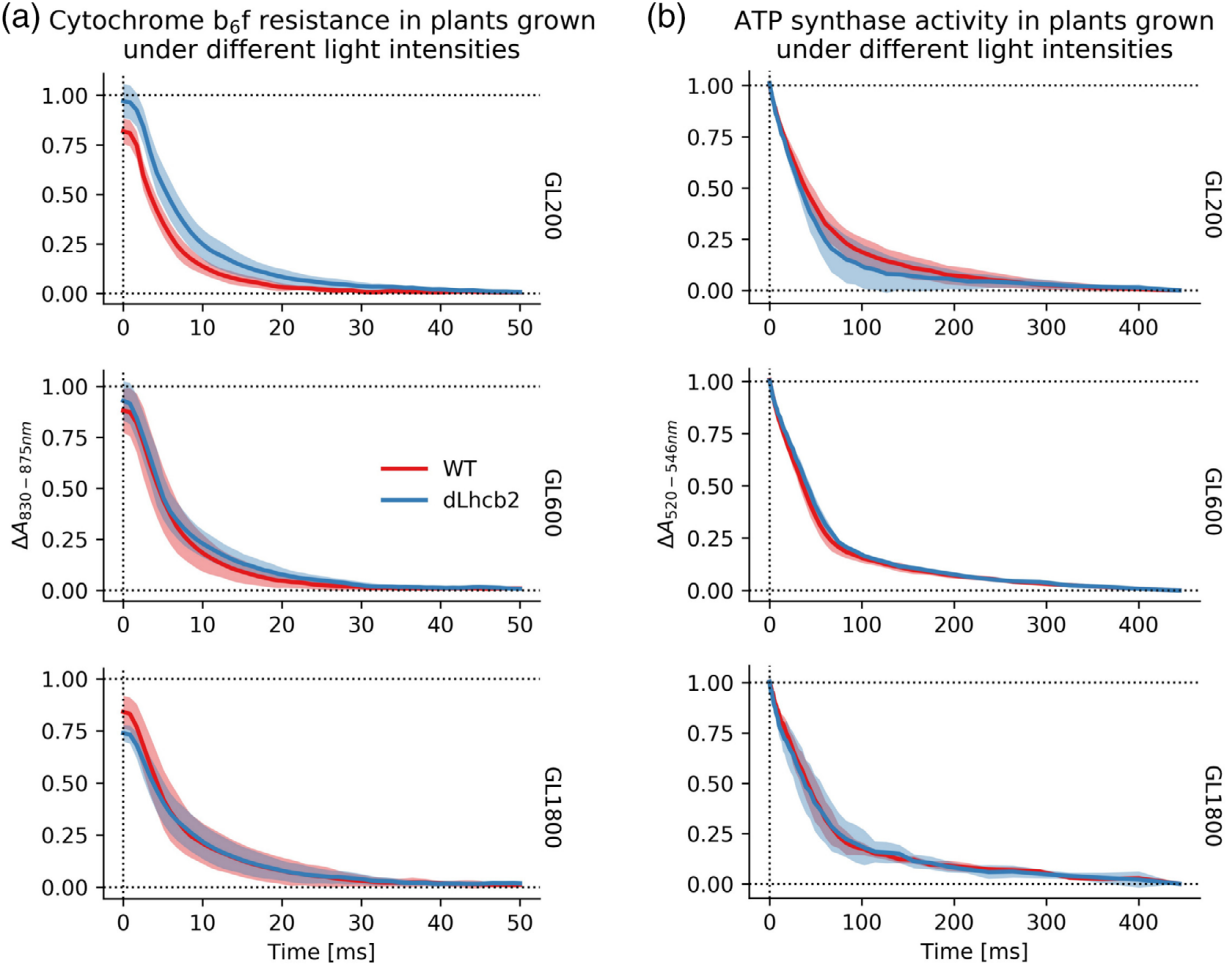
Resistance of cytochrome b_6_f and ATP synthase conductivity. (a) The resistance of the cytochrome b_6_f was measured by the P700^+^ re‐reduction (absorption at 830–875 nm), during the light‐to‐dark transition. Absorption changes were measured on intact leaves, coming from five different plants (*n* = 5) of WT and *dLhcb2* (red and blue traces, respectively), grown under growth light (GL)200, GL600, and GL1800 (respectively the top, middle, and bottom panels). Traces were normalized to the minimum at 50 ms of decay and rescaled to the maximum absorption change measured at the beginning of the measurement (details in the Section 2). Shadows show the standard deviation of the measurements. (b) ATP synthase activity was followed by measuring the ECS decay kinetics (at 520 and 546 nm) after a 50‐s red light illumination on leaves coming from five different plants (*n* = 5) of WT and *dLhcb2* (red and blue traces, respectively) grown under GL200, GL600, and GL1800 (respectively the top, middle, and bottom panels). Traces were double normalized (details in the Section 2). Shadows show the standard deviation of measurements [Color figure can be viewed at https://wileyonlinelibrary.com]

To determine if the large changes in the relative ratio of cytochrome b_6_f and PSI observed during acclimation in the *dLhcb2* mutant had functional consequences for the EF, we measured the cytochrome b_6_f resistance. This was done by following P700^+^ re‐reduction during a light‐to‐dark transition (Figure 7a). The starting absorption level was slightly different, especially in the dLhcb2 grown under different light intensities. However, the kinetics of WT and *dLhcb2* plants was the same for all growth conditions. We verified this hypothesis by fitting the traces with mono‐exponential decay function (Figure S2A) and comparing the values of the decay parameter (Figure S2B). Additionally, we have checked the *P700* ^*red*^ after illumination under different AL intensities (Figure S3). We can conclude that differences in the amount of cytochrome b_6_f between WT and mutant did not influence the EF.

### 3.10 ATP synthase activity

To assess the effect of the changes in the amount of ATP synthase relative to PSI in the *dLhcb2* plants, we measured the ATP synthase conductivity by monitoring the dissipation of the pmf by following the disappearance of the ECS signal during a light‐to‐dark transition (Figure 7b). During illumination, the proton and ion gradients across the thylakoid membrane create a pmf that is released through the ATP synthase (Cruz et al., 2001; Witt, 1979). The ECS decays did not differ between the WT and *dLhcb2*, and between different GL intensities.

### 3.11 Short‐term responses (irradiance curve)

Next, we investigated the photosynthetic and photoprotective capacities of plants fully acclimated to specific GL intensities, challenging them to reach their performance limits by subjecting them for a short time to a range of different light intensities (10 steps in low light [LL]–high light [HL] range).

**Figure 8:**
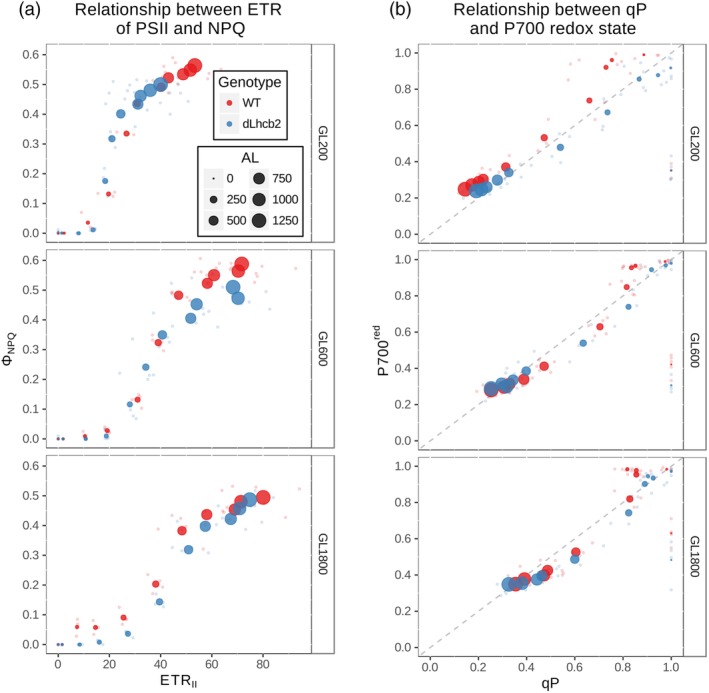
Short‐term responses. (a) Changes in Φ_NPQ_ as a function of ETR_II_ for WT and *dLhcb2* plants (red and blue lines and dots) grown under GL200, GL600, and GL1800 (top, middle, and bottom panel, respectively). All measured points are shown as clouds of small dots. Averaged points for each specific AL (*n* = 3) shown as larger dots. (b) Changes of *P700* ^*red*^ as a function of *qP*. Other details as in the previous panel. The dotted line shows the linear relationship between *qP* and *P700* ^*red*^. AL, actinic light; GL, growth light; NPQ, nonphotochemical quenching [Color figure can be viewed at https://wileyonlinelibrary.com]

First, to compare the EF threshold needed for NPQ activation, we followed the changes of NPQ as a function of EF through PSII (Figure 8a). For NPQ and EF, we monitored *Φ*_*NPQ*_ and *ETR*_*II*_, respectively. To get slightly closer to the reality, instead of using the simplified equation for *ETR*_*II*_ (e.g., White & Critchley, 1999), we performed two additional corrections: (a) for the light interception by PSI and PSII, we used the relative values of PSI/PSII RC ratio from the biochemical data. On the basis of Hogewoning et al. (2012), we assumed 0.73 as the absolute ratio for WT GL200; (b) we used the relative interception area of PSII (from the functional antenna size normalized to WT GL200) as a multiplicative scaling factor.

The kinetics was always sigmoidal as there is an EF threshold level at which ΔpH is large enough or the pH in the lumen is low enough to trigger NPQ. The *ETR*_*II*_ value necessary to trigger NPQ increased for plants grown at higher light intensities.

When *dLhcb2* was grown under 200 or 600 μmol photons m^−2^ s^−1^, the EF threshold of NPQ was similar to that of the WT. Only for HL‐grown plants, the threshold of the mutant was shifted towards higher *ETR*_*II*_ compared with the WT.

We also investigated the changes in the redox state of both photosystems (*qP* and *P*700^*red*^) as a function of light intensity (Figure 8b). This relationship is affected by the light interception area of both PSs, the EF through cytochrome b_6_f and the rate of the PSI acceptor side re‐reduction. Both *qP* and *P*700^*red*^ decreased as a function of AL. As expected, we observed a close to linear relationship between *qP* and *P*700^*red*^, with the higher values of both parameters for plants grown under higher light. Additionally, especially in WT grown under lower light, a deviation from linearity was observed in favour of larger *P*700^*red*^ values at very low AL intensities: *qP* already decreased, whereas *P*700^*red*^ remained high, this was not the case in the mutant. In general, in *dLhcb2*, the linear relationship was maintained, suggesting a comparable EF through both PSs.

## 4 DISCUSSION

Antenna truncation has been proposed as a strategy to increase crop productivity because it would allow a better light distribution in the canopy (Melis, 2009; Ort et al., 2011; Ort et al., 2015). Although this is a promising strategy, it remained to be seen if the reduction of the antenna had negative effects on the functionality and acclimation capacity of the photosynthetic apparatus. Indeed, most of the truncated antenna mutants analysed so far show secondary effects due to a lack of connectivity between the remaining antennae and the core or decreased photoprotection, which counterbalanced the positive effects (van Oort et al., 2010; Miloslavina et al., 2011; Ramel et al., 2013; Dall'Osto et al., 2014). These data clearly show that the absence of some of the minor antenna complexes or of the complete peripheral antenna pool do not represent viable solutions. Mutants showing a partial reduction of the antenna were also analysed (Kirst et al., 2017; Kirst et al., 2018; Slattery et al., 2017). Some of those (Kirst et al., 2017; Kirst et al., 2018) showed an increase in biomass production, whereas others did not (Slattery et al., 2017). These mutants were generated with an untargeted approach (Kirst et al., 2017; Slattery et al., 2017) or targeting a protein that affect chloroplast import (Kirst et al., 2018), which results in the downregulation of several chloroplast components (e.g., Kawata & Cheung, 1990). These results suggest that a more targeted approach, which permits a better control of the changes in chloroplast proteins can be advantageous. For example, the selective reduction of the peripheral antenna complexes (LHCII), the absence of which should not influence excitation energy transfer efficiency to the core, seems to be a promising target. To validate this hypothesis, in this work, we have focused on a mutant in which the Lhcb1 and Lhcb2 pool is only decreased. These proteins are the main components of the LHCIIs; the peripheral antennae of PSII and their amount are modulated when plants are acclimated to high light (Bielczynski et al., 2016; Kouřil et al., 2013; Park, Chow, & Anderson, 1997; Wientjes, Van Amerongen, & Croce, 2013a). The negative effects mentioned above should thus be limited in this mutant. However, to understand how the photosynthetic apparatus responds to the antenna truncation and especially how the mutant plants acclimate to different light conditions, it is crucial to evaluate the viability of this strategy. For example, it was shown previously that a reduction in Lhcb1 and Lhcb2 was counterbalanced by an increase of the minor antenna CP26 (Ruban et al., 2003), thus limiting the effectiveness of the antenna truncation.

The data show that Lhcb1 and Lhcb2 in the mutant are decreased by 60% respectively to the WT. At variance with previous results (Ruban et al., 2003), we did not observe any increase in the level of other antenna complexes (CP26 and Lhcb3) that could compensate for the lack of these two proteins. Surprisingly, under standard conditions, the plants did not respond to the decrease in the absorption cross‐section of the photosystems by increasing the Chl concentration, which instead remained lower than in the WT. The light‐harvesting capacity of the mutants is thus smaller than that of the WT.

The data also show that the photochemical balance between PSI and PSII was maintained (Table 1; Figure S4) although the PSI/PSII ratio did not change compared with the WT under standard conditions. This suggests that the reduction of Lhcb1 and Lhcb2 in the mutant mainly affected the “extra” LHCII pool, which acts as an antenna of both PSI and PSII (Wientjes, Drop, et al., 2013; Wientjes, van Amerongen, & Croce, 2013b). A reduction of this pool leads to a simultaneous reduction of the absorption cross‐section of both photosystems.

**Table 1:** General characterization. Basic fluorescence parameters were measured on the same plants. The NPQ value was measured after 8 min illumination with 1,000 μmol photons m^−2^ s^−1^. The quantification was performed based on three to five replicas

	WT	dLhcb2
Chl *a*/*b* ratio	3.23 ± 0.02^a^	4.00 ± 0.07
Chl/car ratio	3.90 ± 0.02^a^	3.75 ± 0.04
Vio	2.75 ± 0.03^a^	3.02 ± 0.02
Chls/fresh weight (mg g^−1^)	0.86 ± 0.12^a^	0.67 ± 0.05
F_V_/F_M_	0.81 ± 0.01	0.79 ± 0.02
NPQ	2.09 ± 0.19	1.51 ± 0.10

Abbreviations: Chl, chlorophyll; NPQ, nonphotochemical exciton quenching; Vio, violaxanthin.

a Marked with an asterisk, the WT data were taken from Bielczynski et al. (2016).

**Table 2:** Quantification of PSII antenna proteins. Based on the Gaussian fit performed on TT‐SDS PAGE IOD profiles of isolated thylakoids from WT and *dLhcb2* (as shown in Figure 1), we estimated the amount of CP24, CP26, Lhcb1 + 2, and Lhcb3. The amount of antennae was normalized to the amount of CP29. The quantification was performed based on three or five rounds of PAGE (for *dLhcb2* and WT, respectively) with four technical replicates each. With triple asterisks are shown statistically significant groups which means they are different from the WT, based on the post hoc Tukey HSD test

	WT	dLhcb2
CP24	1.19 ± 0.21	1.35 ± 0.34
CP26	1.15 ± 0.14	1.25 ± 0.22
Lhcb1 + 2	5.47 ± 0.46	3.22 ± 0.31***
Lhcb3	1.83 ± 0.27	1.27 ± 0.32***

Abbreviations: Lhc, light‐harvesting complex; PAGE, polyacrylamide gel electrophoresis; IOD, Integrated Optical Density; PSII, Photosystem II; SDS, sodium dodecyl sulphate; TT, Tris‐Tricine; WT, Wild Type.

*** statistical significance at *p* < .001.

**Table 3:** Pigment analysis of WT and *dLhcb2* grown under different light conditions. Total chlorophylls (Chls) were quantified by fitting the absorption spectra of individual pigments to the spectrum of the 80% acetone extracts from a leaf from five different plants (*n* = 5) grown under GL200, 600, and 1800, and normalized to the fresh weight. The Chl *a*/*b* ratio and chlorophyll/carotenoid (Chl/Car) ratio were determined in the same way from isolated thylakoid membranes in three repetitions (*n* = 3). The same extracts were used for the quantification by HPLC of the carotenoids: neoxanthin (Neo), violaxanthin (Vio), lutein (Lut), and β‐carotene (β‐Car). All carotenoids were calculated per 100 Chls

	WT^a^			dLhcb2		
	GL200	GL600	GL1800	GL200	GL600	GL1800
Chls/fresh weight (mg g^−1^)	0.86 *±* 0.12	0.78 *±* 0.03	0.39 *±* 0.10	0.67 *±* 0.05	0.56 *±* 0.06	0.39 *±* 0.08
Chl *a*/*b* ratio	3.23 *±* 0.02	3.25 *±* 0.01	3.56 *±* 0.05	4.00 *±* 0.07	3.99 *±* 0.03	4.06 *±* 0.07
Chl/Car ratio	3.90 *±* 0.02	3.70 *±* 0.01	3.23 *±* 0.05	3.75 *±* 0.04	3.75 *±* 0.01	2.90 *±* 0.02
Neo	3.42 *±* 0.05	3.74 *±* 0.03	4.00 *±* 0.18	2.79 *±* 0.01	2.88 *±* 0.02	3.98 *±* 0.15
Vio	2.75 *±* 0.03	3.03 *±* 0.00	4.11 *±* 0.18	3.02 *±* 0.02	3.39 *±* 0.04	5.62 *±* 0.13
Lut	12.36 *±* 0.012	12.90 *±* 0.04	13.98 *±* 0.28	5.62 *±* 0.07	11.96 *±* 0.10	15.86 *±* 0.39
*β*‐Car	6.68 *±* 0.02	6.69 *±* 0.07	7.59 *±* 0.18	9.02 *±* 0.18	8.41 *±* 0.21	9.03 *±* 0.74
Cars	25.6 *±* 0.2	27.0 *±* 0.1	31.0 *±* 0.5	26.7 *±* 0.3	26.6 *±* 0.1	34.5 *±* 0.2

Abbreviations: HPLC, High Performance Loquid Chromatography; WT, Wild Type.

a The WT data were taken from Bielczynski et al. (2016).

Interestingly, although in the mutant, the PSI/PSII ratio was the same as in the WT, the amounts of both cytochrome b_6_f and ATP synthases decreased. These complexes are crucial for maintaining linear EF and for the photoprotective regulation of the photosynthetic apparatus (reviewed in Colombo et al., 2016). The reduction of both complexes in the mutant seems thus to be a strategy to maintain the photosynthetic control at the level of the cytochrome b_6_f in the presence of a smaller absorption cross‐section of the photosystems. The lower amount of ATP synthase permits a slower dissipation of ΔH^+^ across the membrane (reviewed in Schöttler et al., 2015) and as a consequence enables the chloroplasts to keep high levels of NPQ.

Because the reduction of Lhcb1 and Lhcb2 is used by plants to adapt to increasing light conditions, it was expected that the composition of the photosynthetic membrane of the WT in high light became similar to that of the mutant. The data show that indeed the Chl concentration in HL is the same in WT and mutant. However, although the functional antenna size of PSII is smaller in the WT in HL compared with LL, it is still larger than that of the mutant. This result suggests the existence of a minimum PSII antenna size and that the antenna size of the mutant is just below this level. However, this does not seem to have major consequences under most light conditions, and it is only when plants are grown in very high light (GL1800) that a decrease in PSI and an increase in lutein are observed. These effects are probably compensating for a higher light sensitivity of PSII.

In conclusion, our results show that the photosynthetic light phase is a robust system easily optimized to reach homeostasis under different light conditions thanks to the structural and the functional flexibility of the photosynthetic apparatus. The overlap of several acclimation responses to the light is an important evolutionary adaptation, as the lack of one (e.g., reduction of the antenna size) can be easily compensated by the other (modulation of the stoichiometry of the photosynthetic complexes in the thylakoid membrane).

**Figure 9:**
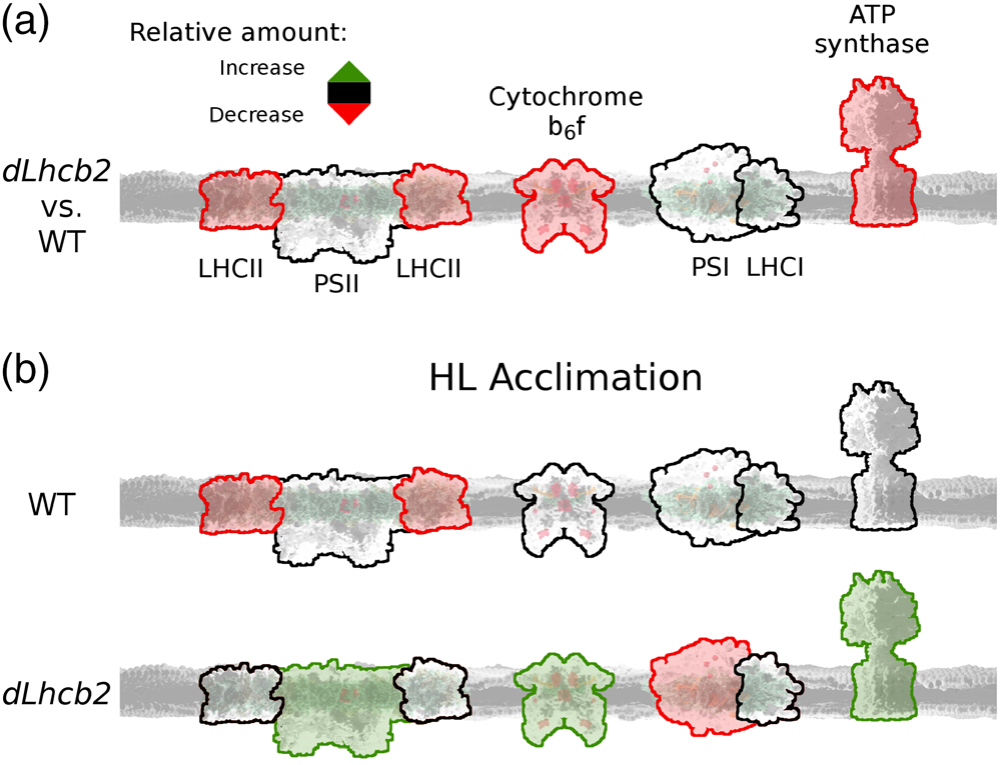
Summary of thylakoid membrane remodelling. (a) Differences in the number of light phase complexes between *dLhcb2* and WT (PSII, PSI, cytochrome b_6_f, and ATP synthase, respectively) grown under GL200. The relative increase/decrease of the amount of a specific complex (or part of the complex) was marked by a green or red contour line, respectively. No change was marked as a black contour line. (b) Changes occurring in the thylakoid membrane during HL acclimation (GL1800) in *dLhcb2* and WT (top and bottom, respectively). PSI, Photosystem I; PSII, Photosystem II [Color figure can be viewed at https://wileyonlinelibrary.com]

Our analysis shows that the reduction of Lhcb1 and Lhcb2 has no negative effects on the function of the photosynthetic apparatus, thanks to a change in the level of the other components of it (Figure 9); and equally important, it does not induce compensatory effects by increasing the expression of other pigment‐proteins complexes. The partial reduction of Lhcb1 and Lhcb2 is thus a viable strategy to produce robust plants with truncated antenna size.

## Supporting information


**Appendix** S1. Supporting Information
