# Supplementary material for: Consequences of the reduction of the Photosystem II antenna size on the light acclimation capacity of Arabidopsis thaliana
Source: Plant Cell Environ. 2020 Feb 5;43(4):866–79. doi: 10.1111/pce.13701 (PMC7154682; doi:10.1111/pce.13701)
Supplement: Supplementary file 1 — Appendix S1. Supporting Information [file PCE-43-866-s001.pdf]

Supplemental data

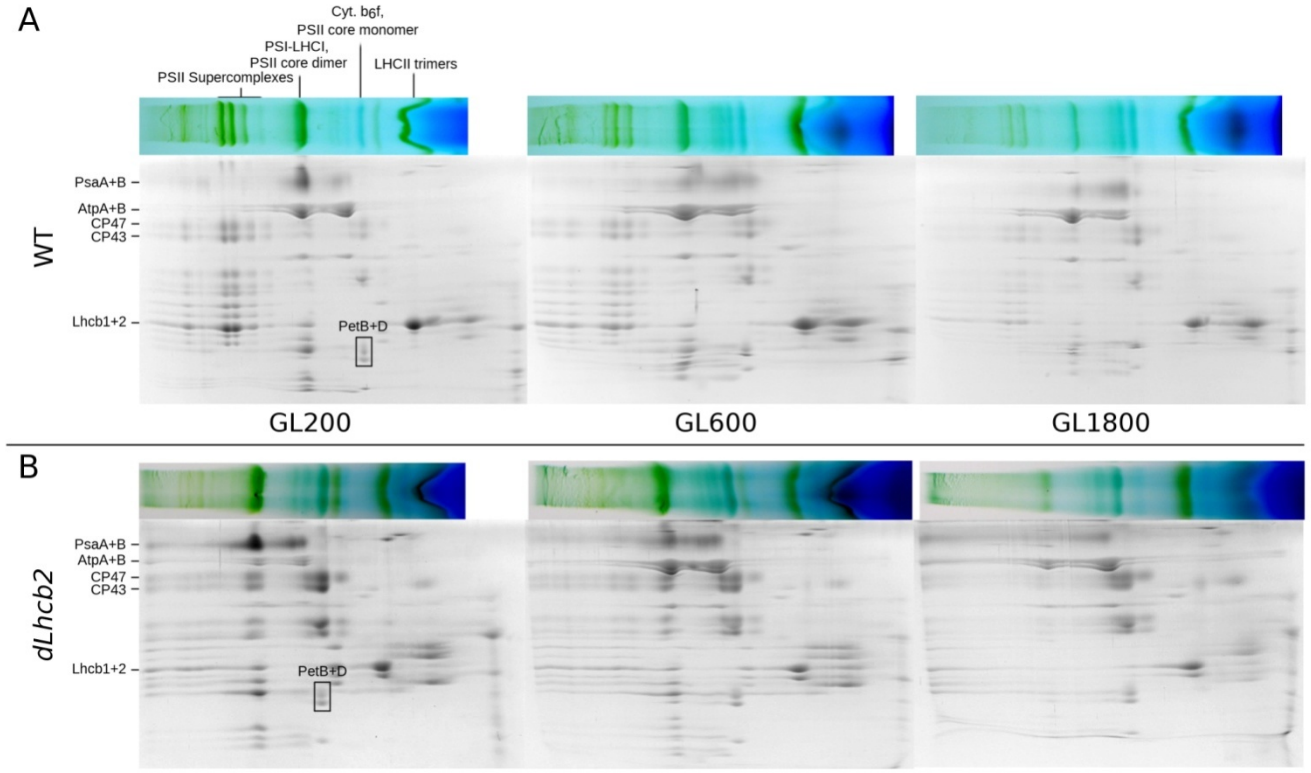

Supplemental Figure 1. 2D-PAGE analysis of WT and dLhcb2 grown under different light intensities. 2D-PAGE was performed on thylakoid membranes from the WT and *dLhcb2* (A and B panels, respectively) grown under GL200, 600 and 1800 (from left to right, respectively). On the top of each denaturating gel, the strip excised from a BN-PAGE is shown. On the side, the labels corresponding to specific groups of protein dots are indicated.

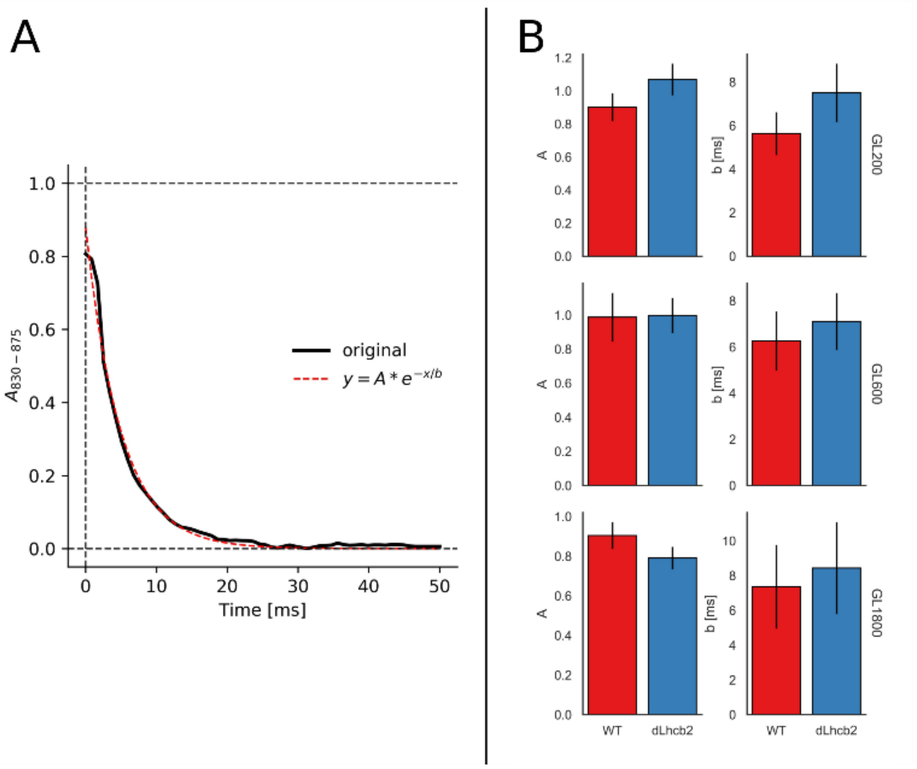

Supplemental Figure 2. Exponential fit of the cytochrome b_6f resistance measurement. (A) An Example of a trace of absorption measurement (black) fitted with a mono-exponential decay (red). (B) Histograms reporting the amplitude and time (left and right column, respectively)obtained for the fitting of WT and *dLhcb2* traces (red and blue bars, respectively) grown under GL200, 600 and 1800 (from top to bottom panels, respectively). The measurements were performed on 5 different plants. The standard deviation is reported.

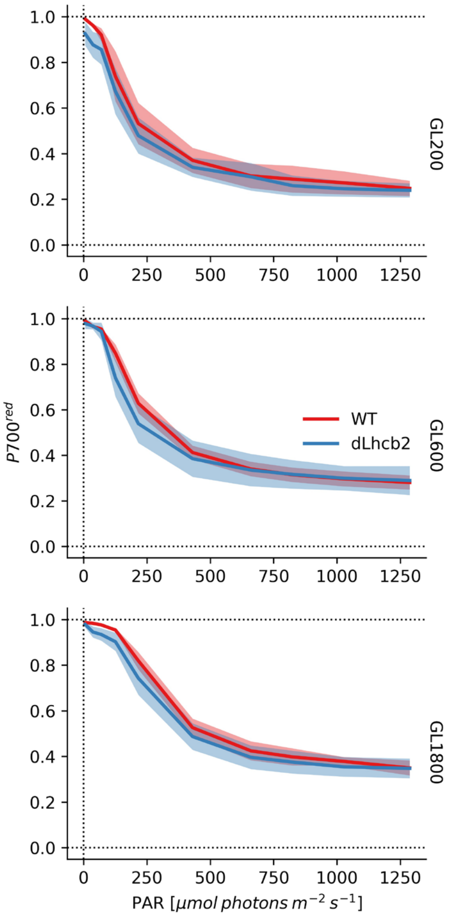

Supplemental Figure 3. *$P700^{red}$* light curve analysis of WT and dLhcb2 grown under different light intensities. *$P700^{red}$* light curve analysis was performed on WT and *dLhcb2* (red and blue trace, respectively) grown under GL200, 600 and 1800 (from top to bottom panels, respectively). A mean and the standard deviation from measurements performed on 3-5 different plants are shown as a solid trace and its shadow, respectively.

PSII functionality in the mutant

A double pulse experiment was performed to check if the reduction of the PSII antenna size influences the linear electron flow (LEF), by comparing the reduction and oxidation kinetics of the electron transfer chain components in the mutant and the WT.

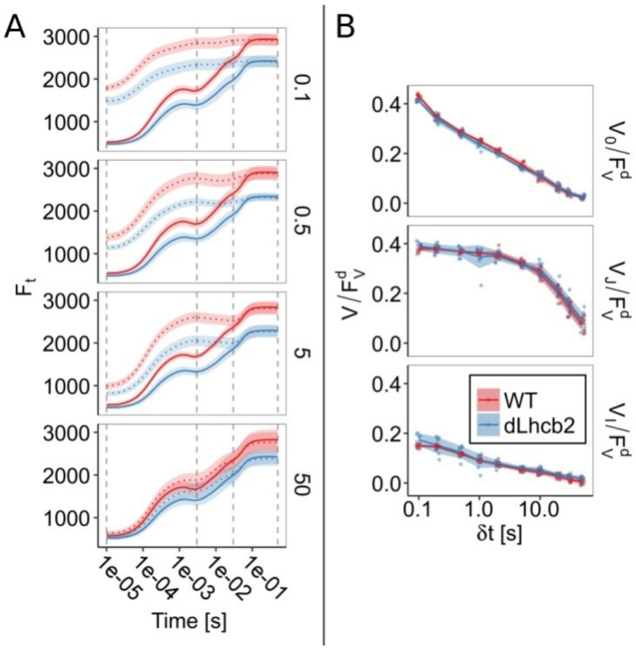

Supplemental Figure 4. PSII reoxidation rate. (A) Fluorescence induction kinetics after two consecutive 1 s long SPs (first pulse solid line and second pulse dashed) were measured on intact leaves, coming from 10 different plants (n = 10) of WT and *dLhcb2* (red and blue traces, respectively), grown under GL200. The time between pulses ranges between 0.1 ms and 50 ms (respectively from the top to bottom panels). The gray dashed lines indicate the O, J, I and P steps (from left to right). (B) The relationship between variable fluorescence between two consecutive SPs at a specific phase of fluorescence induction V_0, V_J and V_I (marked respectively with three first gray dashed lines in panel A), and the time between the pulses (δt; from top to bottom panel, respectively). Variable fluorescence was normalized to the extent of fluorescence in darkness (F_V^d).

During a standard fluorescence rise, we differentiate between the earliest fluorescence intensity (F_0 or O) at 10 μs of the 0.5 s long SP, the peak time-point (F_M or P) at the end, and two other inflection time-points at 3 ms and 300 ms (J and I steps, respectively).

In a double pulse experiment, we modulate the time between two consecutive pulses to observe the regeneration kinetics of the original rise kinetics. Increasing the time interval between the pulses, allows us to monitor the re-oxidation of the electron transport components as a function of the dark interval's time. The dark-kinetics of O follow the Q_A^- re-oxidation (Schansker et al., 2005; Schansker et al., 2011). The kinetics consist of a fast drop (halftime of below a second) followed by two slower phases. The recombination can occur through two different pathways (reviewed in Petrouleas and Crofts, 2005). The first phase is mostly caused by the recombination *via* the donor side of PSII. However, as this type of recombination can occur only when the oxygen-evolving complex of the PSII is in S_2 or S_3 state, the re-oxidation of the rest of Q_A^- depends on a partial re-oxidation of the PQ pool.

The JI phase reflects the reduction of the PQ-pool (Schreiber et al., 1989; Schansker et al., 2005; Schansker el., 2011). The regeneration of the J step depends on the re-oxidation of the PQ-pool through the plastoquinol oxidase activity of the plant, and the exchange in the Q_B-site of a reduced PQ for an oxidized one (Tóth et al., 2007). The recovery of the I-step reflects the re-oxidation of the Fd and FeS clusters in the acceptor side of PSI (Schansker et al., 2005).

Compared to the WT, the mutant's fluorescence was slightly decreased. However, the decrease might come from the lower pigment content of the mutant (Chls/fresh weight in Table 1). As for the re-oxidation kinetics (Figure 2B), the mutant did not differ in any respect from the WT.

References:

Schansker G., Tóth S.Z. & Strasser R.J. (2005) Methylviologen and dibromothymoquinone treatments of pea leaves reveal the role of photosystem I in the Chl a fluorescence rise OJIP. Biochimica et biophysica acta 1706, 250–61.

Schansker G., Tóth S.Z., Kovács L., Holzwarth A.R. & Garab G. (2011) Evidence for a fluorescence yield change driven by a light-induced conformational change within photosystem II during the fast chlorophyll a fluorescence rise. Biochimica et Biophysica Acta - Bioenergetics 1807, 1032–1043.

Petrouleas V. & Crofts A.R. (2005) The Iron-Quinone Acceptor Complex. In The Light-Driven Water:Plastoquinone Oxidoreductase. pp. 177–206. Springer-Verlag, Berlin/Heidelberg.

Society R. & Sciences B. (2017) Devices and Methods for Room-Temperature Fluorescence Analysis Author (s): U . Schreiber , C . Neubauer and C . Klughammer Source : Philosophical Transactions of the Royal Society of London . Series B , Biological Sciences , Vol . 323 , No . 1216 , New Vistas in Measurement of Photosynthesis (Apr . 12 , 1989), Published by : Royal Society Stable URL : http://www.jstor.org/stable/2396763. 323, 241–251.

Tóth S.Z., Schansker G., Garab G. & Strasser R.J. (2007) Photosynthetic electron transport activity in heat-treated barley leaves: The role of internal alternative electron donors to photosystem II. Biochimica et Biophysica Acta - Bioenergetics 1767, 295–305.
